# Periodic repolarization dynamics as predictor of risk for sudden cardiac death in chronic heart failure patients

**DOI:** 10.1038/s41598-021-99861-1

**Published:** 2021-10-15

**Authors:** Saúl Palacios, Iwona Cygankiewicz, Antoni Bayés de Luna, Esther Pueyo, Juan Pablo Martínez

**Affiliations:** 1 BSICoS Group, Aragón Institute of Engineering Research, IIS Aragón, Universidad de Zaragoza, Zaragoza, Spain; 2 Department of Electrocardiology, Medical University of Lodz, Lodz, Poland; 3 Cardiovascular Research Foundation, Cardiovascular ICCC-Program, Research Institute Hospital de la Santa Creu i Sant Pau, IIB-Sant Pau, Barcelona, Spain; 4 CIBER en Bioingeniería, Biomateriales y Nanomedicina (CIBER-BBN), Zaragoza, Spain

**Keywords:** Heart failure, Cardiomyopathies, Predictive markers, Cardiomyopathies, Heart failure, Risk factors

## Abstract

The two most common modes of death among chronic heart failure (CHF) patients are sudden cardiac death (SCD) and pump failure death (PFD). Periodic repolarization dynamics (PRD) quantifies low-frequency oscillations in the T wave vector of the electrocardiogram (ECG) and has been postulated to reflect sympathetic modulation of ventricular repolarization. This study aims to evaluate the prognostic value of PRD to predict SCD and PFD in a population of CHF patients. 20-min high-resolution (1000 Hz) ECG recordings from 569 CHF patients were analyzed. Patients were divided into two groups, $\hbox {PRD}^+$ and $\hbox {PRD}^-$, corresponding to PRD values above and below the optimum cutoff point of PRD in the study population. Univariate Cox regression analysis showed that SCD risk in the $\hbox {PRD}^+$ group was double the risk in the $\hbox {PRD}^-$ group [hazard ratio (95% CI) 2.001 (1.127–3.554), $\hbox {p}<0.05$]. The combination of PRD with other Holter-based ECG indices, such as turbulence slope (TS) and index of average alternans (IAA), improved SCD prediction by identifying groups of patients at high SCD risk. PFD could be predicted by PRD only when combined with TS [hazard ratio 2.758 (1.572–4.838), $\hbox {p}<0.001$]. In conclusion, the combination of PRD with IAA and TS can be used to stratify the risk for SCD and PFD, respectively, in CHF patients.

## Introduction

Approximately 1–2% of the adult population in Western societies is diagnosed with heart failure (HF)^1,2^, including over 10% of people aged 70 years or older^3^. An estimated 64 million people suffer from HF worldwide and the total associated cost in health programs is estimated to reach $400 billion by 2030^4^. HF is a clinical syndrome accompanied by a high burden of co-morbidities^5^ and poor prognosis^6^.

In chronic HF (CHF) patients, sudden cardiac death (SCD) represents the cause of death in 30–50% of them^7^ and accounts for more than 60% of all cardiovascular deaths out of hospital^8^. SCD is defined as death due to unexpected circulatory arrest that occurs within an hour of the onset of symptoms or during sleep^9^. Another common cause of death in CHF is pump failure death (PFD), resulting from progressive pump failure. Treatment with $\beta$-blockers and implantable cardioverter defibrillators (ICDs) are effective therapies for prevention of SCD, improving the quality of life of affected patients and altering the mode of death from SCD to PFD^10^. Based on this evidence, there is an important need to successfully predict the mode of death in CHF, which could contribute to a more cost-effective use of medications or devices.

A variety of non-invasive indices derived from resting electrocardiograms (ECGs) or ambulatory Holter recordings have been proposed for stratifying CHF patients according to their PFD and SCD risk. Among others, heart rate variability (HRV)^11^, QT interval variability index (QTVi)^12^, baroreflex sensitivity^13^, fragmented QRS^14,15^, T-wave alternans^16,17^ or turbulence slope^18^ have been investigated. Periodic repolarization dynamics (PRD) has been proposed to assess sympathetic modulation of ventricular repolarization by measuring low-frequency (LF, below 0.1 Hz) oscillations in the T-wave vector^19^. Elevated PRD has been related to increased arrhythmic risk in a range of cardiac diseases and conditions^19–22^.

Autonomic imbalance, with increased sympathetic activity and withdrawal of vagal activity, has been described as a hallmark of HF^23^. Such an imbalance leads to worsening of prognosis in CHF patients^24^, with higher likelihood for PFD and SCD^25,26^. On this basis, we hypothesize that, if the PRD index is a marker of sympathetic-associated repolarization instability, it could be used for risk prediction in a CHF population. This work specifically aims to assess the capacity of the PRD index, calculated by using an update of the originally proposed algorithm^27^, for SCD and PFD risk stratification of CHF patients. PRD was analyzed both individually and in combination with other repolarization and autonomic-related ECG indices to predict SCD and PFD.

## Results

The study population consisted of 569 CHF patients (409 men and 160 women), aged 20–80 years (mean $63 \pm 12$), enrolled in the MUSIC (*MUerte Subita en Insuficiencia Cardiaca*) study. From the 992 included in the original MUSIC study^28^, 341 patients had atrial fibrillation, flutter or pacemaker and were excluded for the present analysis. Out of the remaining 651 patients, 82 patients did not have an available high-resolution ECG recording and were therefore not included. Mean left ventricular ejection fraction (LVEF) was 37.0 ± 13.8% (range 12–70%), with most patients (55%) presenting $\hbox {LVEF}\le 35\%$. Half of the patients (50.1%) had ischemic cardiomyopathy and 259 patients had previous myocardial infarction (45.5%). Detailed characteristics of the study population are shown in Table 1.

**Table 1:** Clinical and ECG variables of database.

**Clinical variables**	
Age (years)	64 (16)
Gender (male)	409 (71.9%)
NYHA class III	99 (17.4%)
LVEF $\le 35\%$	312 (54.8%)
Ischemic etiology	285 (50.1%)
Diabetes mellitus	212 (37.3%)
Amiodarone	53 (9.3%)
$\hbox {NT-proBNP}>1000 \,\hbox {pg/mL}$	186 (32.7%)
Prior MI	259 (45.5%)
**ECG variables**	
Median RR (ms)	857 (179)
RR range (ms)	697 (279)
QRS > 120 ms	234 (41.1%)
NSVT and VPB > 240 in 24-h	148 (26.0%)
$\hbox {IAA} \ge 3.7 \,\upmu \hbox {V}$	139 (24.4%)
$\hbox {TS} \le 2.5 \,\hbox {ms/RR}$	249 (43.8%)

Data are represented as median (interquartile range) for continuous variables and as number (percentage) for dichotomized variables.

*IAA* index of average alternans, *LVEF* left ventricular ejection fraction, *NSVT* nonsustained ventricular tachycardia, *NYHA* New York Heart Association, *TS* turbulence slope, *VPB* ventricular premature beat.

### Association of PRD with cardiac events

PRD was dichotomized according to the optimum cutoff point obtained from ROC analysis for each of the investigated endpoints. Optimum cutoff values were 1.33$^{\circ }$ for SCD, 1.31$^{\circ }$ for PFD (approximately coincident with the median PRD over the study population) and 1.32$^{\circ }$ for cardiac death (CD), comprising SCD and PFD. In each case, $\hbox {PRD}^+$ and $\hbox {PRD}^-$ subgroups were defined to contain patients with PRD values above and below the cutoff point, respectively. Additional information on the derivation of the cutoff values is provided in “Methods” section.

**Figure 1:**
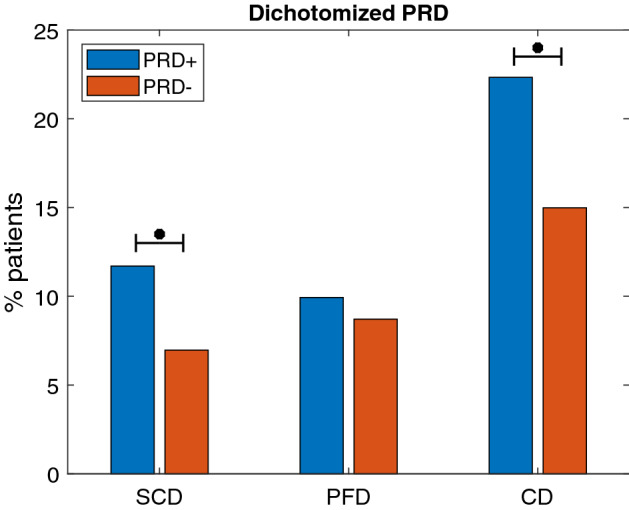
Percentages of SCD, PFD and CD victims in $\hbox {PRD}^+$ and $\hbox {PRD}^-$ groups. $^{*}\hbox {p}<0.05$.

The percentages of SCD, PFD and CD victims in $\hbox {PRD}^+$ and $\hbox {PRD}^-$ groups are presented in Fig. 1. There were 106 CD victims in the study population (18.6%), of which 53 died of SCD and 53 of PFD. The SCD mortality rate was significantly higher (p = 0.018) in the $\hbox {PRD}^+$ group (33 victims, 11.7%) than in the $\hbox {PRD}^-$ group (20 victims, 7%). Regarding PFD, no significant differences in mortality rates between $\hbox {PRD}^+$ and $\hbox {PRD}^-$ groups were found (28 victims, 10% in $\hbox {PRD}^+$ vs 25 victims, 8.7% in $\hbox {PRD}^-$). For CD, significant differences were found, with mortality being remarkably higher in the $\hbox {PRD}^+$ group as compared to the $\hbox {PRD}^-$ group (22.3% vs 15%).

### SCD and PFD risk prediction based on PRD and other individual variables

**Figure 2:**
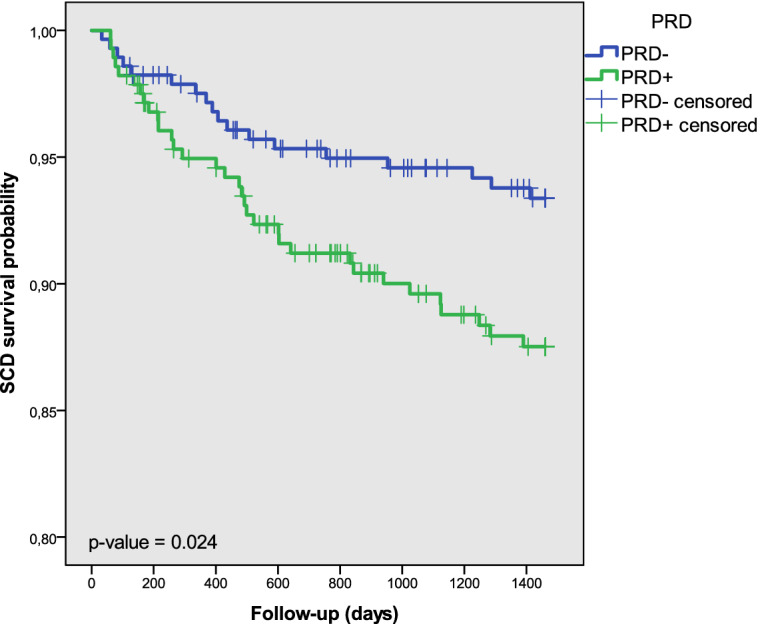
Estimated probability curve of SCD survival for $\hbox {PRD}^+$ and $\hbox {PRD}^-$ groups.

Kaplan–Meier survival analysis showed that $\hbox {PRD}^+$ patients had significantly lower SCD survival probability than $\hbox {PRD}^-$ patients ($p=0.024$), as illustrated in Fig. 2. When accounting for PFD as a competing risk, Fine and Gray analysis^29^ showed that the cumulative SCD incidence curves for $\hbox {PRD}^+$ and $\hbox {PRD}^-$ patients were statistically significantly different ($\hbox {p} = 0.048$). When PFD was considered as endpoint, no significant differences in the survival rates were found between $\hbox {PRD}^+$ and $\hbox {PRD}^-$ groups.

Univariate Cox analysis results for SCD risk prediction by the PRD index as well as by demographical, clinical and other ECG variables are shown in Table 2. The variables significantly associated with SCD were New York Heart Association (NYHA) class III, LVEF $\le 35\%$, NSVT and $\hbox {VPB} > 240$, N-terminal prohormone of brain natriuretic peptide $(\hbox {NT-proBNP})>1000 \,\hbox {pg/mL}$, $\hbox {IAA}^+$, $\hbox {TS}^+$ and $\hbox {PRD}^+$. None of the HRV variables (LFn, HFn or LF/HF) was associated with SCD risk. Regarding PFD, $\hbox {PRD}^+$ was not able to predict this endpoint, whereas age, ischemic etiology, prior myocardial infarction, NYHA class, $\hbox {NT-proBNP}>1000 \,\hbox {pg/mL}$ and $\hbox {TS}^+$ were significant predictors (Table 3).

**Table 2:** Univariate Cox analysis for SCD as endpoint.

	Univariate	
	HR (95%)	p value
**Endpoint: SCD**		
Age	1.016 (0.992–1.040)	0.198
Gender	1.967 (0.960–4.030)	0.064
Ischemic etiology	1.630 (0.940–2.827)	0.082
Prior MI	1.639 (0.952–2.823)	0.074
NYHA class III	2.370 (1.318–4.263)	0.004**
LVEF$\le 35\%$	2.231 (1.227–4.056)	0.009**
LFn	2.198 (0.556–8.687)	0.261
HFn	0.455 (0.115–1.799)	0.261
LF/HF	1.080 (0.998–1.168)	0.055
NSVT and $\hbox {VPB}>240$	2.167 (1.255–3.743)	0.006**
Diabetes mellitus	1.378 (0.800–2.372)	0.248
$\hbox {NT-proBNP}>1000 \,\hbox {pg/mL}$	2.339 (1.349–4.056)	0.002**
$\hbox {IAA}^+$	2.312 (1.318–4.055)	0.003**
$\hbox {TS}^+$	2.619 (1.418–4.838)	0.002**
$\hbox {PRD}^+$	2.001 (1.127–3.554)	0.018*

Age, gender, ischemic etiology, prior myocardial infarction, NYHA class, LVEF, HRV indices (LFn, HFn, LF/HF), combined NSVT and $$\hbox {VPB}>240$$, diabetes, $$\hbox {NT-proBNP}>1000 \,\hbox {pg/mL}$$, $$\hbox {IAA}^+$$, $$\hbox {TS}^+$$ and $$\hbox {PRD}^+$$ were the analyzed variables. $$^{*}\hbox {p}<0.05$$, $$^{**}\hbox {p}<0.01$$.

**Table 3:** Univariate Cox analysis for PFD as endpoint.

	Univariate	
	HR (95%)	p value
**Endpoint: PFD**		
Age	1.051 (1.024–1.079)	$1.9\times 10^{-4}$ ***
Gender	1.135 (0.616–2.090)	0.685
Ischemic etiology	1.946 (1.110–3.411)	0.020*
Prior MI	1.941 (1.119–3.365)	0.018*
NYHA class III	2.715 (1.524–4.836)	0.001**
$\hbox {LVEF}\le 35\%$	1.743 (0.987–3.077)	0.056
LFn	0.802 (0.219–2.939)	0.739
HFn	1.247 (0.340–4.570)	0.739
LF/HF	0.957 (0.846–1.083)	0.488
NSVT and $\hbox {VPB}>240$	1.738 (0.991–3.047)	0.054
Diabetes mellitus	1.903 (1.110–3.260)	0.019$^{*}$
$$\hbox {NT-proBNP}>1000 \,\hbox {pg/mL}$$	4.945 (2.692–9.082)	$$2.6\times 10^{-7}$$ $$^{***}$$
$\hbox {IAA}^+$	1.115 (0.594–2.093)	0.735
$$\hbox {TS}^+$$	4.964 (2.477–9.947)	$$6\times 10^{-6}$$ $$^{***}$$
$\hbox {PRD}^+$	1.242 (0.724–2.129)	0.431

Age, gender, ischemic etiology, prior myocardial infarction, NYHA class, LVEF, HRV indices (LFn, HFn, LF/HF), combined NSVT and $$\hbox {VPB}>240$$, diabetes, $$\hbox {NT-proBNP}>1000 \,\hbox {pg/mL}$$, $$\hbox {IAA}^+$$, $$\hbox {TS}^+$$ and $$\hbox {PRD}^+$$ were the analyzed variables. $$^{*}\hbox {p}<0.05$$, $$^{**}\hbox {p}<0.01$$, $$^{***}\hbox {p}<0.001$$.

Multivariate Cox proportional hazards regression model for SCD prediction including clinical variables such as the combination of NSVT and $\hbox {VPB} > 240$, $\hbox {LVEF} \le 35\%$, NYHA class and $\hbox {NT-proBNP}>1000 \,\hbox {pg/mL}$ together with $\hbox {PRD}^+$ led to the results presented in Table 4. $\hbox {PRD}^+$ and some of the clinical variables were independent predictors of SCD, being associated with similar hazard ratio (HR) values. When considering PFD as a competing risk event, the regression model for SCD as endpoint included clinical variables and PRD, the latter with a HR of 1.65, even if being only marginally predictive ($\hbox {p} = 0.095$).

**Table 4:** Multivariate SCD risk prediction including the following variables: NYHA class, $\hbox {LVEF}\le 35\%$, combined NSVT and $\hbox {VPB}>240$, $\hbox {NT-proBNP}>1000 \,\hbox {pg/mL}$ and $\hbox {PRD}^+$.

	Multivariate	
	HR (95%)	p value
**Endpoint: SCD**		
NYHA class III	1.606 (0.850–3.036)	0.144
$\hbox {LVEF}\le 35\%$	2.034 (1.050–3.940)	0.035*
NSVT and $\hbox {VPB}>$240	1.516 (0.840–2.734)	0.167
$\hbox {NT-proBNP}>1000 \,\hbox {pg/mL}$	1.632 (0.908–2.933)	0.101
$\hbox {PRD}^+$	1.825 (1.019–3.268)	0.043*

$$^{*}\hbox {p}<0.05$$.

When tested in the subpopulation of patients with CHF of non-ischemic etiology, PRD was able to predict SCD in the multivariate model ($\hbox {HR} = 2.497$, $\hbox {p} = 0.05$). In the subpopulation of patients with CHF of ischemic etiology, PRD was not predictive of SCD in the univariate model and therefore it was not included in a multivariate model.

We finally tested the capacity of $\hbox {PRD}^+$ for CD prediction. The HRs for univariate and multivariate Cox analyses were 1.64 ($\hbox {p} = 0.014$) and 1.63 ($\hbox {p} = 0.016$), respectively. Other demographical, clinical and ECG variables were also able to predict CD in a univariate Cox model (Table 5).

**Table 5:** Univariate Cox analysis for SCD and PFD victims (CD as endpoint).

	Univariate	
	HR (95%)	p value
**Endpoint: CD**		
Age	1.032 (1.014–1.050)	$$3.6\times 10^{-4}$$ $$^{***}$$
Gender	1.461 (0.921–2.319)	0.108
Ischemic etiology	1.780 (1.201–2.636)	$$0.004^{**}$$
Prior MI	1.783 (1.211–2.624)	$$0.003^{**}$$
NYHA class	2.537 (1.681–3.830)	$$9\times 10^{-6}$$ $$^{***}$$
$$\hbox {LVEF}\le 35\%$$	1.965 (1.303–2.965)	$$0.001^{**}$$
LFn	1.304 (0.508–3.344)	0.581
HFn	0.767 (0.299–1.968)	0.581
LF/HF	1.030 (0.963–1.102)	0.384
NSVT and $$\hbox {VPB}>240$$	1.944 (1.315–2.874)	$$0.001^{**}$$
Diabetes mellitus	1.620 (1.106–2.372)	$0.013^{*}$
$$\hbox {NT-proBNP}>1000 \,\hbox {pg/mL}$$	3.330 (2.227–4.979)	$$4.6\times 10^{-9}$$ $$^{***}$$
$\hbox {IAA}^+$	1.637 (1.083–2.474)	$0.019^{*}$
$$\hbox {TS}^+$$	3.552 (2.251–5.604)	$$5\times 10^{-9}$$ $$^{***}$$
$\hbox {PRD}^+$	1.636 (1.104–2.425)	$0.014^{*}$

Age, gender, ischemic etiology, prior myocardial infarction, NYHA class, LVEF, HRV indices (LFn, HFn, LF/HF), combined NSVT and $$\hbox {VPB}>240$$, $$\hbox {NT-proBNP}>1000\,\hbox {pg/mL}$$, $$\hbox {IAA}^+$$, $$\hbox {TS}^+$$ and $$\hbox {PRD}^+$$ were the variable to analyze in this model. $$^{*}\hbox {p}<$$0.05, $$^{**}\hbox {p}<0.01$$, $$^{***}\hbox {p}<0.001$$.

If rather than dichotomizing PRD based on optimum cutoff points, dichotomization was performed according to the median PRD over the whole study population, results only slightly changed. In particular, the HR for SCD prediction using $\hbox {PRD}^+$ decreased from 2.001 to 1.934 for univariate Cox analysis and from 2.002 to 1.934 for multivariate Cox analysis.

### SCD and PFD risk prediction based on the combination of PRD with other variables

**Figure 3:**
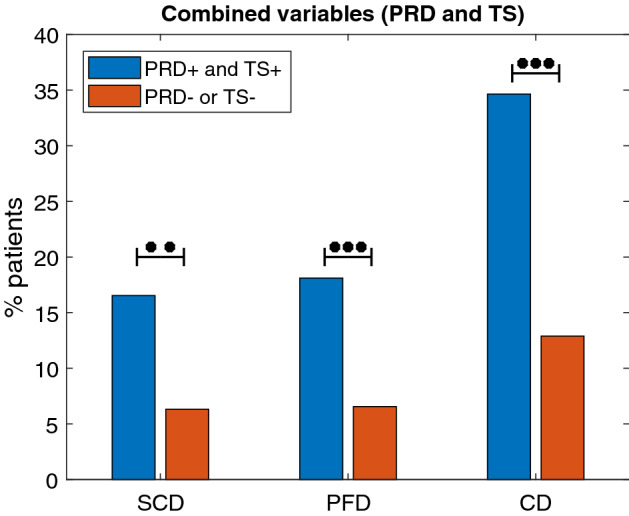
Percentages of SCD, PFD and CD victims in the $$\hbox {PRD}^+$$ and $$\hbox {TS}^+$$ group and in the rest of patients. $$^{**}\hbox {p}<0.01$$, $$^{***}\hbox {p}<0.001$$.

**Figure 4:**
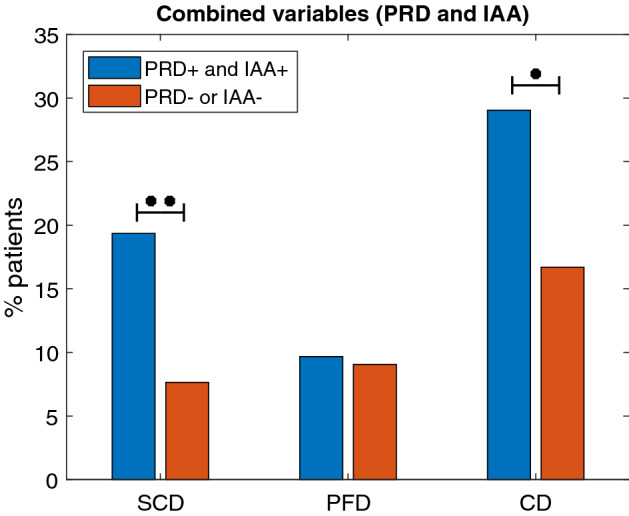
Percentages of SCD, PFD and CD victims in the $$\hbox {PRD}^+$$ and $$\hbox {IAA}^+$$ group and the rest of patients. $$^{*}\hbox {p}<0.05$$, $$^{**}\hbox {p}<0.01$$.

To improve the predictive power for SCD risk, the combination of PRD with other Holter-based variables, such as IAA and TS, was assessed. As shown in Fig. 3, patients with $\hbox {PRD}^+$ and $\hbox {TS}^+$ had significantly higher SCD mortality ($\hbox {p} < 0.05$) and PFD mortality ($\hbox {p} < 0.001$) than the rest of patients. CD mortality was significantly higher too. Results for the combination of PRD and IAA are presented in Fig. 4. Patients with $\hbox {PRD}^+$ and $\hbox {IAA}^+$ had increased SCD mortality ($\hbox {p} = 0.007$) and CD mortality ($\hbox {p} = 0.023$) than the rest of the population. No significant differences were found with respect to PFD mortality ($\hbox {p} = 0.816$).

**Figure 5:**
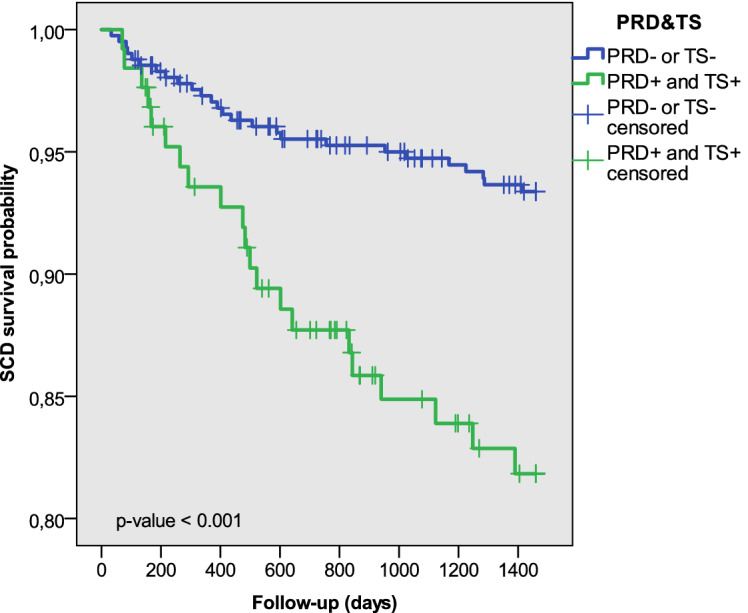
Estimated probability curve of SCD for two subgroups defined by $\hbox {PRD}^+$ and $\hbox {TS}^+$.

**Figure 6:**
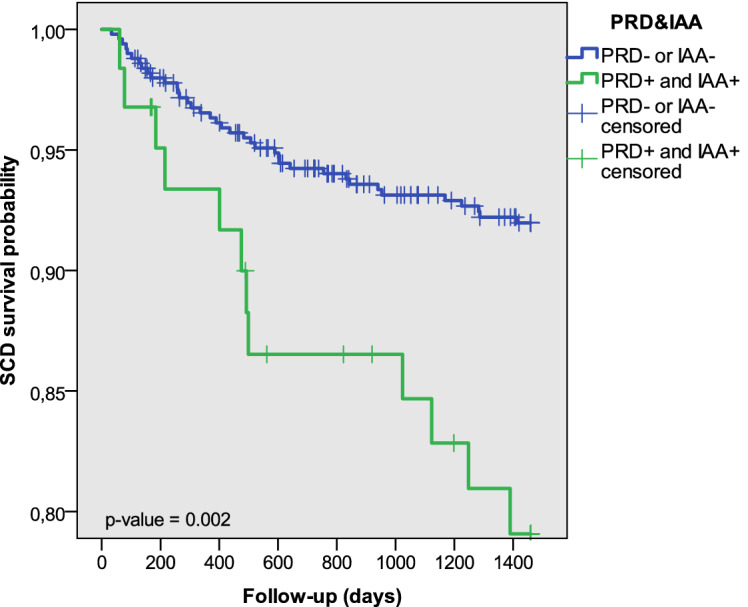
Estimated probability curve of SCD for two subgroups defined by $\hbox {PRD}^+$ and $\hbox {IAA}^+$.

Figures 5 and 6 show Kaplan–Meier probability curves for SCD survival using the combined variables $\hbox {PRD}^+$ and $\hbox {TS}^+$ and $\hbox {PRD}^+$ and $\hbox {IAA}^+$, respectively. For the two variables, significantly lower survival was found for patients with positive PRD and TS or IAA. When PFD was considered as endpoint, differences in survival probabilities were only statistically significant when using $\hbox {PRD}^+$ and $\hbox {TS}^+$. Considering PFD as a competing risk event for SCD, Fine and Gray analysis showed significantly higher cumulative SCD incidence for $\hbox {PRD}^+$ and $\hbox {TS}^+$ ($\hbox {PRD}^+$ and $\hbox {IAA}^+$, respectively) patients than the rest of the population ($\hbox {p} < 0.001$ in all cases). When accounting for SCD as a competing risk event for PFD, the cumulative PFD incidence for patients with $\hbox {PRD}^+$ and $\hbox {TS}^+$ was significantly higher than for the rest of patients in the population ($\hbox {p} < 0.001$).

**Table 6:** Univariate Cox analysis results for $\hbox {PRD}^+$ and $\hbox {TS}^+$, $\hbox {PRD}^+$ and $\hbox {IAA}^+$, $\hbox {PRD}^+$ and $\hbox {TS}^+$ and $\hbox {PRD}^+$ and $\hbox {IAA}^+$ separately considering SCD, PFD or both as endpoints.

	Univariate	
	HR (95%)	p value
**Endpoint: SCD**		
$$\hbox {PRD}^+$$ and $$\hbox {TS}^+$$	3.090 (1.729–5.522)	$$1.4\times 10^{-4} \, ^{***}$$
$$\hbox {PRD}^+$$ and $$\hbox {IAA}^+$$	2.803 (1.465–5.364)	0.002$$^{**}$$
**Endpoint: PFD**		
$$\hbox {PRD}^+$$ and $$\hbox {TS}^+$$	2.758 (1.572–4.838)	$$4\times 10^{-4} \, ^{***}$$
$\hbox {PRD}^+$ and $\hbox {IAA}^+$	0.763 (0.275–2.117)	0.603
**Endpoint: CD**		
$$\hbox {PRD}^+$$ and $$\hbox {TS}^+$$	3.089 (2.066–4.617)	$$3.8\times 10^{-8} \, ^{***}$$
$\hbox {PRD}^+$ and $\hbox {IAA}^+$	1.888 (1.134–3.142)	0.015$^{*}$

$$^{*}\hbox {p}<0.05$$, $$^{**}\hbox {p}<0.01$$, $$^{***}\hbox {p}<0.001$$.

In univariate Cox analysis (Table 6) with SCD as endpoint, $\hbox {PRD}^+$ and $\hbox {TS}^+$ as well as $\hbox {PRD}^+$ and $\hbox {IAA}^+$ were risk predictors, with associated HRs of 3.1 and 2.8, respectively. For PFD as endpoint, $\hbox {PRD}^+$ and $\hbox {TS}^+$ patients presented more than two and a half times higher risk than the rest of the population. For CD as endpoint, $\hbox {PRD}^+$ and $\hbox {TS}^+$ was associated with a HR of 3.1, while $\hbox {PRD}^+$ and $\hbox {IAA}^+$ with a HR of 1.9.

**Table 7:** Multivariable SCD risk prediction including the following variables: NYHA class, $\hbox {LVEF}\le 35\%$, combined NSVT and $\hbox {VPB}>240$, $\hbox {NT-proBNP}>1000 \,\hbox {pg/mL}$ and a combined ECG variable that can be either $\hbox {PRD}^+$ and $\hbox {TS}^+$ or $\hbox {PRD}^+$ and $\hbox {IAA}^+$.

	Multivariate		Multivariate	
	HR (95%)	p value	HR (95%)	p value
**Endpoint:SCD**				
NYHA class III	1.586 (0.813–3.092)	0.176	1.858 (0.987–3.497)	0.055
LVEF$\le 35\%$	2.084 (1.028–4.225)	0.042$^{*}$	2.228 (1.136–4.371)	0.020$^{*}$
NSVT and $\hbox {VPB}>240$	1.278 (0.679–2.405)	0.447	1.552 (0.861–2.798)	0.144
$\hbox {NT-proBNP}>1000 \,\hbox {pg/mL}$	1.560 (0.831–2.930)	0.167	1.807 (0.997–3.275)	0.051
$\hbox {PRD}^+$ and $\hbox {TS}^+$	1.998 (1.068–3.738)	0.030*	–	–
$$\hbox {PRD}^+$$ and $$\hbox {IAA}^+$$	–	–	3.046 (1.578–5.877)	0.001$$^{**}$$

$$^{*}\hbox {p}<0.05$$, $$^{**}\hbox {p}<0.01$$.

Results from multivariate Cox proportional hazard regression analysis including NYHA class, LVEF $\le 35\%$, NSVT and $\hbox {VPB}>240$ and $\hbox {NT-proBNP}>1000 \,\hbox {pg/mL}$ as well as one of the two combined ECG variables at a time are presented in Table 7 for SCD as endpoint. Both $\hbox {PRD}^+$ and $\hbox {IAA}^+$ and $\hbox {PRD}^+$ and $\hbox {TS}^+$ independently predicted SCD risk with HRs of 3.0 and 2.1, respectively. Multivariate regression analysis for SCD with PFD as competing risk indicated that the combined variable $\hbox {PRD}^+$ and $\hbox {IAA}^+$ ($\hbox {PRD}^+$ and $\hbox {TS}^+$, respectively) predicted SCD independently of other clinical variables, with an associated HR of 2.9 (2.4, respectively, being $\hbox {p} < 0.01$ in all cases).

In the subpopulation of patients with CHF of non-ischemic etiology, $\hbox {PRD}^+$ and $\hbox {TS}^+$ predicted SCD with a HR 3.2 (p = 0.012) and PFD with a HR of 3.43 ($\hbox {p} = 0.011$) independently of other clinical variables. $\hbox {PRD}^+$ and $\hbox {IAA}^+$ predicted SCD risk in both ischemic and non-ischemic etiology subpopulations, with associated HR of 3.3 and 2.9, respectively.

To complete the study, we tested the capacity of the combined variables for CD risk prediction. Table 8 shows the results for the two tested multivariable proportional hazard models. Both $\hbox {PRD}^+$ and $\hbox {IAA}^+$ and $\hbox {PRD}^+$ and $\hbox {TS}^+$ predicted CD risk independently of other demographic and clinical variables with HRs of 2.4 and 2.0, respectively.

**Table 8:** Multivariable CD risk prediction including the following variables: age, ischemic etiology, prior myocardial infarction, NYHA class, $\hbox {LVEF}\le 35\%$, combined NSVT and $\hbox {VPB}>240$, $\hbox {NT-proBNP}>1000 \,\hbox {pg/mL}$ and a combined ECG variable that can be either $\hbox {PRD}^+$ and $\hbox {TS}^+$ or $\hbox {PRD}^+$ and $\hbox {IAA}^+$.

	Multivariate		Multivariate	
	HR (95%)	p value	HR (95%)	p value
**Endpoint: CD**				
Age	1.015 (0.995–1.035)	0.135	1.021 (1.001–1.041)	0.035$^{*}$
Ischemic etiology	1.515 (0.776–2.956)	0.224	2.038 (1.058–3.923)	0.033$^{*}$
Prior MI	1.009 (0.523–1.946)	0.980	0.947 (0.500–1.791)	0.866
NYHA class	1.771 (1.119–2.801)	0.015$^{*}$	1.914 (1.226–2.988)	0.004$^{*}$
LVEF$\le 35\%$	1.568 (0.977–2.517)	0.062	1.727 (1.089–2.737)	0.020$^{*}$
NSVT and $\hbox {VPB}>240$	1.236 (0.789–1.937)	0.354	1.489 (0.969–2.287)	0.069
$$\hbox {NT-proBNP}>1000 \,\hbox {pg/mL}$$	2.092 (1.326–3.302)	0.002$$^{**}$$	2.293 (1.478–3.557)	2$$\times 10^{-4} \, ^{***}$$
$$\hbox {PRD}^+$$ and $$\hbox {TS}^+$$	1.893 (1.215–2.947)	0.005$$^{**}$$	–	–
$$\hbox {PRD}^+$$ and $$\hbox {IAA}^+$$	–	–	2.431 (1.446–4.085)	0.001$$^{**}$$

$$^{*}\hbox {p}<0.05$$, $$^{**}\hbox {p}<0.01$$, $$^{***}\hbox {p}<0.001$$.

## Discussion

This work investigates the performance of PRD, characterizing the oscillatory behavior of the T wave, for cardiac risk stratification in a population of CHF patients. Although PRD has been previously used in various contexts^19,27,30–34^, in this study, we computed PRD in a large CHF cohort to predict the risk of the two most common modes of death, namely SCD and PFD, both considering PRD on its own and in combination with other ECG-based indices.

Our results show that CHF patients presenting high PRD values have nearly twice higher risk of suffering SCD than CHF patients with low PRD. These findings are in agreement with previous clinical studies showing a relationship between increased PRD and enhanced mortality, particularly due to ventricular arrhythmias, in different patient populations^19,35^. Specifically in post-myocardial infarction patients with impaired LVEF, elevated PRD has been related to enhanced risk for SCD^20^. Also, PRD has been suggested to be potentially useful in guiding clinical decisions on prophylactic implantation of ICDs in patients with ischemic and non-ischemic cardiomyopathy^36^.

PRD measures the magnitude of LF oscillations in the T wave vector of the ECG and has been postulated to reflect sympathetic modulation of ventricular repolarization^19^. The same range of LF oscillations in the T wave vector accounted for by PRD has been reported for the action potential duration (APD) in in vivo studies on HF patients^37^. Through in silico simulations, synergistic $\beta$-adrenergic and mechanical effects induced by sympathetic activation have been shown to contribute to the generation of these LF oscillations of APD^38,39^. Those results have been supported by subsequent experimental studies showing that increased sympathetic activity potentiates such oscillations and $\beta$-adrenergic blockade attenuates them^37,40^. In the presence of calcium overload and/or reduced repolarization reserve, both being commonly associated with HF, the amplitude of LF oscillations of APD have been theoretically shown to be magnified, facilitating the occurrence of arrhythmogenic events^39,41^. A recent in vivo study in a canine model of ventricular remodeling caused by chronic atrioventricular block has provided evidence that dogs inducible for ventricular arrhythmias present higher LF oscillations of repolarization than non-inducible dogs^42^. Based on all these observations, the results of our study possibly suggest that CHF patients with high PRD present augmented repolarization variability that can lead to destabilization of repolarization and promote arrhythmogenesis.

Since CHF is a complex clinical syndrome, some studies have reported the benefit of using combined ECG risk markers and/or a risk score integrating information from various clinical and ECG variables to improve clinical decision making^43–47^. The use of markers providing information about different pathophysiological processes associated with CHF has been shown to be very useful^48,49^. Here, a combination of PRD and other ECG-based indices are tested for SCD and PFD risk prediction. For PFD prediction, the combination of PRD with TS, measuring heart rate turbulence, stratifies the study population into two groups according to the risk of PFD. The risk of dying from PFD is two and a half times higher in patients with high PRD ($\hbox {PRD}^+$) and low TS ($\hbox {TS}^+$) than in the rest of patients. Nevertheless, the capacity for PFD prediction of TS on its own is already very good, as reported in previous studies^50^, and PRD only slightly improves it. TS represents a vagally-mediated response of heart rate to ventricular premature beats with involvement of baroreflex sensitivity. TS has been considered an excellent marker of neurohormonal activation in CHF patients^50,51^. Since autonomic dysfunction and neurohormonal activation have a relevant role in the progression of CHF, our results on the capacity for PFD risk stratification of combined PRD and TS support assessment of such CHF landmarks noninvasively from short-term ECGs (for PRD evaluation) and Holter recordings (for TS evaluation). In terms of SCD prediction, the stratifying ability of PRD is enhanced when combined with the marker IAA evaluating T-wave alternans amplitude over 24 h. While T-wave alternans, measured by IAA in this work, is related to abnormal cardiac function^17^, PRD measures changes in sympathetic modulation of ventricular repolarization^19^. The synergistic information provided by the two variables serves to improve SCD prediction. In all the above described findings, the risk stratification capacity of PRD is maintained when accounting for competing risk events in survival analyses.

NT-proBNP concentration, dichotomized using the threshold (1000 pg/mL) proposed in the original MUSIC study^28^, was not a significant predictor of SCD in multivariable regression models comprising PRD or combined ECG markers. When the endpoint was CD, $\hbox {NT-proBNP}>1000 \,\hbox {pg/mL}$ could significantly predict CD in a multivariable model. Other studies have proposed alternative thresholds for NT-proBNP, like $\hbox {NT-proBNP}>5180 \,\hbox {pg/mL}$^52^, and found the dichotomized variable to be a predictor of both SCD and PFD. However, as our CHF population includes only patients in NYHA classes II and III, only 7% of the patients had NT-proBNP values above 5180 pg/mL.

On the basis of previous studies showing that PRD is unrelated to HRV and respiratory activity^19^, we hypothesized that PRD and HRV markers could add complementary information. However, our results show that HRV markers do not have a relationship either with SCD or PFD and, thus, we have not combined them with PRD for risk stratification purposes. On the other hand, of the clinical variables assessed in our study, NYHA class and LVEF $\le 35\%$ are the two with the highest HR for both PFD and SCD. In any case, the HR of these two clinical variables are lower than those obtained for the combined ECG variables PRD and TS in the prediction of PFD and PRD and IAA in the prediction of SCD. LVEF is widely used in the clinics to identify high-risk patients but its accuracy is low^53^. Regarding NYHA class, as it reflects a subjective assessment and can change frequently over short periods of time^28^, its interpretation is more critical than other variables. The analysis conducted in this work suggests that the adjunct use of clinical variables and ECG-based markers can improve the prognostic value of all of them and render a risk score with remarkably superior performance to predict the two most common modes of death in CHF patients.

An important aspect regarding the use of PRD is the fact that it can be measured from 5-min ECGs by using the phase-rectified signal averaging (PRSA)-based method employed in this study, first described by Rizas et al.^35^ and subsequently updated by Palacios et al.^27^ Here, we use 20-min ECGs, from which the 5-min segment associated with minimum PRD is selected. This is an advantage as compared to a number of previously analyzed markers, such as IAA^17^, QTVi^12^, TS^54^, $\Delta \alpha ^{QT}$^55^, $\Delta \alpha ^{Tpe}$^56^ or TMR^57^, which require longer ECG recordings or specific protocols, like stress tests or steady heart rate, for their evaluation. While the combination of PRD with other Holter-based markers improves risk stratification, it requires long-duration recordings. On this basis, we propose that PRD could be used in an initial step to select patients with high CD risk. Over those selected patients, the use of longer duration signals and associated variables could be useful to specifically predict each of the death modes. Several works have proposed other markers related to repolarization variability measured from short-term ECG recordings, such as the variance normalized by the mean of QT end, QT peak or T-peak-to-T-end (Te) intervals, and have evaluated them in CHF populations. In recent studies, the mean and/or standard deviation of Te have been shown to predict 30-day mortality^58,59^ and mortality in hospital^60^ among decompensated CHF patients.

To sum up, our study documents the prognostic value of PRD in combination with other Holter-derived markers to predict PFD and SCD in a large cohort of CHF patients. Future studies on CHF cohorts including a larger number of SCD and PFD victims would allow conducting more robust statistical analysis to confirm the findings here reported. Also, studies on larger populations would facilitate assessing the capacity of PRD for risk stratification in specific CHF patient subpopulations, such as those with reduced or preserved LVEF or at different NYHA classes, among others.

## Methods

### Study population

The MUSIC study is a prospective, multicenter, longitudinal study designed to assess risk predictors of cardiac mortality and SCD in ambulatory patients with CHF. Patients were consecutively enrolled from the specialized CHF clinics of eight University Hospitals between April 2003 and December 2004. All had symptomatic CHF (NYHA class II–III) and were treated according to institutional guidelines. The study included patients with either depressed or preserved LVEF. Patients with preserved LVEF were included if they had CHF symptoms and a prior hospitalization for HF or some objective signs of HF confirmed by chest X-ray (findings of pulmonary congestion) and/or echocardiography (abnormal LV filling pattern and LV hypertrophy). Patients were excluded if they had recent acute coronary syndrome or severe valvular disease amenable for surgical repair. Patients with other concomitant diseases expected to reduce life-expectancy were also excluded. All patients gave written informed consent and the study protocol was approved by all the institutional investigation and ethics committees from the following participant hospitals: Valme Hospital, Santiago de Compostela Hospital, Son Dureta Hospital, Arrixaca Hospital, Gregorio Marañon Hospital, Joan XXIII Hospital, Insular Las Palmas Hospital, Sant Pau Hospital^28^. All methods were performed in accordance with the relevant guidelines and regulations.

### Study protocol

For each patient, two ECG recordings were available: a 24-h ambulatory ECG sampled at 200 Hz in 3 orthogonal X, Y and Z leads using SpiderView recorders (ELA Medical, SorinGroup, Paris, France) and a prior 20-min ECG sampled at 1000 Hz while patients were resting in supine position. In this study, PRD was measured from the 20-min ECG recording. Patients were followed every 6 months on an outpatient basis for an average of 48 months. Death was defined as SCD if it was: (1) a witnessed death occurring within 60 min from the onset of new symptoms, unless a cause other than cardiac was obvious; (2) an unwitnessed death ($<24\,\hbox {h}$) in the absence of preexisting progressive circulatory failure or other causes of death; or (3) a death during attempted resuscitation. Deaths occurring in hospital as a result of refractory progressive end-stage CHF, or CHF patients undergoing heart transplantation, were defined as PFD. Endpoints were reviewed and classified by the MUSIC Study Endpoint Committee. A description of clinical data for the overall population and more detailed information about the study protocol can be found in Vazquez et al.^28^.

### ECG pre-processing

High-resolution ECG signals were preprocessed by a 50 Hz notch filter to remove powerline interference. QRS complexes were detected using Aristotle software^61^. The heart timing method described in Mateo and Laguna^62^ was applied to detect irregular sinus beats. As the main analysis was focused on the T-wave, a 40-Hz low-pass filter was subsequently applied onto the ECG signals to remove noise without distorting the T-wave shape. Finally, baseline wander was estimated by cubic splines interpolation and cancelled.

For each beat *i*, a window including the T-wave was defined from the QRS position, denoted by $\hbox {QRS}_{{{\text {i}}}}$, and the associated RR interval preceding it, denoted by $\hbox {RR}_{{{\text {i}}}}$. The T-wave window onset, denoted by $\hbox {T}_{\text {on}_{{{\text {i}}}}}$, was set at 90 ms after the $\hbox {QRS}_{{{\text {i}}}}$ mark: $\hbox {T}_{\text {on}_{{{\text {i}}}}} = \hbox {QRS}_{{{\text {i}}}} + 90 \,\hbox {ms}$. The T-wave window end, denoted by $\hbox {T}_{\text {end}_{{{\text {i}}}}}$, was defined as $\hbox {T}_{\text {end}_{{{\text {i}}}}} = \hbox {QRS}_{{{\text {i}}}} + \hbox {min}(360 \,\hbox {ms}, \frac{2}{3} \text {RR}_{{{\text {i}}}}$) for $\hbox {RR}_{{{\text {i}}}}$ below 720 ms. For $\hbox {RR}_{{{\text {i}}}}$ equal or higher than 720 ms, $\hbox {T}_{\text {end}_{{{\text {i}}}}} = \hbox {QRS}_{{{\text {i}}}} + 360 \,\hbox {ms}$.

The noise level was estimated in each lead and beat by measuring the root mean square value of the high-frequency components (above 15 Hz) in a window around the T-wave, similarly to previous works^56^. A global measure of noise for each beat *i* was obtained by summing the noise levels of all leads and denoted by $\hbox {V}^{RMS}_{noise}(i)$. If a beat was too noisy ($\hbox {V}^{RMS}_{noise}>$140 $\upmu$V), the associated T-wave marks ($\hbox {T}_{\text {on}}$ and $\hbox {T}_{\text {end}}$) were discarded for further analysis, but the associated QRS mark was retained.

### PRD computation

PRD was measured in the preprocessed signal by using the method described in Palacios et al.^27^, a modified version of the original method described in Rizas et al.^19^. This method included the following steps:

1. T waves were selected using the T wave windows defined in section ECG pre-processing.
2. A constant value was subtracted from each T wave in each of the analyzed leads so that the amplitude at $\hbox {T}_{{end}}$ was set to 0 mV.
3. The average electrical vector was calculated for each T-wave window. The angle $\hbox {dT}^{\circ }$ between two consecutive T-wave windows was calculated by the dot product of the corresponding average vectors.
4. A 10th-order median filter was used to attenuate outliers and artifacts in the $\hbox {dT}^{\circ }$ time series.

**Figure 7:**
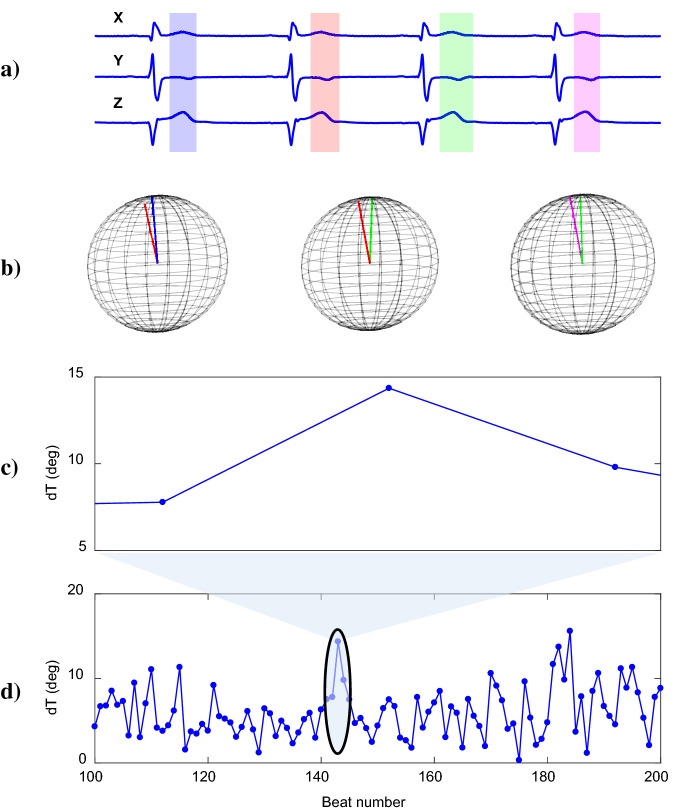
Illustration of steps for PRD calculation from ECG recording in Frank lead configuration (**a**) T-waves for four consecutive beats. (**b**) Three-dimensional visualization of each pair of T-wave vector. (**c**,**d**) Angle between two consecutive T-wave vectors, $\hbox {dT}^{\circ }$, along 100 beats.

Steps (i) to (iii) are illustrated in Fig. 7.

Over the obtained $\hbox {dT}^{\circ }$ time series, a method based on PRSA^63^ was applied to evaluate the oscillations measured by the PRD index^35^:

5. Anchor points were defined by comparing averages of $M=9$ values of the $\hbox {dT}^{\circ }$ series previous and posterior to the anchor point candidate $x_{\text {i}}$. A point $x_{\text {i}}$ was considered to be an anchor point if:
  1 $$\begin{aligned} \frac{1}{M} \sum _{j=0}^{M-1}x_{i+j} > \frac{1}{M} \sum _{j=1}^{M}x_{i-j} \end{aligned}$$
  The value of *M* = 9 was established because it allows detecting frequencies in the range of interest (from 0.025 to 0.1 Hz), as fully described in Bauer et al.^63^.
6. Windows of 2*L* values were defined around each anchor point. Anchor points in the last *L* samples of the $\hbox {dT}^{\circ }$ series were discarded, as windows of length 2*L* could not be defined around them. In this study, *L* = 20 was chosen, as it allowed detection of frequencies in the range of interest.
7. The PRSA series was obtained by averaging the $\hbox {dT}^{\circ }$ values over all 2L-sample windows contained in each 5-min segment.

For each 5-min segment, a PRD value was defined as the difference between the maximum and minimum values of the PRSA series. For the 20-min recording of each subject, a unique PRD value was calculated as the minimum PRD over the analyzed 5-min segments with 4-min overlap.

**Figure 8:**
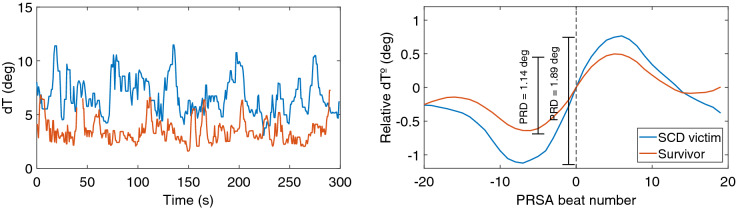
dT series along 300 s (left panel) and PRSA curve (right panel), where anchor beats were set to 0$^{\circ }$ , from a SCD victim and a survivor.

Illustrative examples of $\hbox {dT}^{\circ }$ and PRSA time series from two subjects, a SCD victim and a survivor, are shown in Fig. 8.

By setting a cutoff point of 1.33$^{\circ }$, an optimum thresholds was identified as that maximizing the geometric mean of sensitivity and specificity for SCD as endpoint, two groups were defined: $\hbox {PRD}^+$, containing those patients with PRD above the cutoff value and $\hbox {PRD}^-$, containing the remaining patients. Additionally, the median PRD in the study population was applied rendering a value of 1.31$^{\circ }$. Similarly, the optimum value for CD as endpoint was 1.32$^{\circ }$.

### Heart rate variability analysis

For the 5-min segment with minimum PRD, HRV indices were computed as in Bailón et al.^64^. Power spectral density (PSD) was estimated based on the periodogram. LF and high-frequency (HF) powers were calculated as the areas under the PSD within the 0.04–0.15 Hz and 0.15–0.5 Hz frequency bands, respectively. Normalized powers, denoted by LFn and HFn, were obtained by dividing LF and HF powers by the sum of the two. The ratio between LF and HF powers, denoted by LF/HF, was additionally computed^65^.

### Clinical variables and other Holter-based ECG indices

Clinical variables such as LVEF $\le 35\%$, $\hbox {NT-proBNP} >1000\, \hbox {pg/mL}$, non-sustained ventricular tachycardia (NSVT) and a number of ventricular premature beats (VPB) $> 240$, whose capacity for SCD and PFD risk prediction has been previously shown^28^, were included in our analysis.

Additionally, other Holter-based indices calculated in previous studies of our group were considered based on their risk prediction power. On the one hand, the index of average alternans, IAA, quantifying the average amplitude of T-wave alternans over a 24-h period, was computed by automatic ECG analysis^17^. IAA was shown to risk stratify for SCD in the study population here analyzed when dichotomized at $3.7 \,\upmu$V to define $\hbox {IAA}^+$ and $\hbox {IAA}^-$ groups^17^. On the other hand, turbulence slope, TS, describing the initial phase of sinus rhythm deceleration after a VPB, was determined as the maximum positive slope of a regression line assessed over any of 5 consecutive RR intervals within the first 20 sinus RR intervals after a VPB during the 24-h ECG recording^51^. TS was shown to stratify risk for both SCD and PFD when dichotomized at 2.5 ms/RR, with $\hbox {TS}^+$, denoting the group of patients with TS below the threshold, being associated with higher risk than the group $\hbox {TS}^-$ containing the remaining patients^51^.

### Statistical analysis

Continuous variables are presented as median [interquartile range (IQR)]. The Mann–Whitney U test (or Wilcoxon rank-sum test) was used for univariate comparisons of continuous variables between patient groups. Survival probability was estimated by Kaplan–Meier analysis, with the log-rank test used to assess group differences. Univariate and multivariate Cox^66^ proportional hazards regression models and Fine and Gray analysis for competing risk^29^ were used for prediction of endpoints. Hazard ratios (HR) and 95% confident intervals (CI), expressed as HR [95% CI] were quantified. P values $< 0.05$ were considered for statistical significance. Variables being significantly different between groups in the univariate analysis were input to the multivariate Cox regression model. Data were analyzed using MATLAB R2017a (9.2), SPSS (version 24.0) and R software (version 4.1).

## Conclusions

This study tests PRD, a non-invasive marker of repolarization instability associated with low-frequency oscillations in sympathetic activity, as a predictor of SCD and PFD risk in CHF patients. The combination of PRD with an index of T wave alternans further enhances the capacity of PRD for SCD risk stratification. Additionally, the combination of PRD with heart rate turbulence slope allows predicting PFD risk. Altogether, the value of ECG markers, either derived from short-term ECGs or ambulatory Holter recordings, is highlighted as a means to improve prognosis in CHF patients beyond commonly used clinical variables.

## Data Availability

The current study analyzed datasets which are not publicly available due to restrictions in the ethical permission but the data can be accessed through the corresponding author upon reasonable request and with permission of the MUSIC Study Committee.
